# Balanced cholinergic modulation of spinal locomotor circuits via M2 and M3 muscarinic receptors

**DOI:** 10.1038/s41598-019-50452-1

**Published:** 2019-10-01

**Authors:** Filipe Nascimento, Lennart R. B. Spindler, Gareth B. Miles

**Affiliations:** School of Psychology and Neuroscience, University of St. Andrews, St. Andrews, KY16 9JP United Kingdom

**Keywords:** Motor neuron, Spinal cord

## Abstract

Neuromodulation ensures that neural circuits produce output that is flexible whilst remaining within an optimal operational range. The neuromodulator acetylcholine is released during locomotion to regulate spinal motor circuits. However, the range of receptors and downstream mechanisms by which acetylcholine acts have yet to be fully elucidated. We therefore investigated metabotropic acetylcholine receptor-mediated modulation by using isolated spinal cord preparations from neonatal mice in which locomotor-related output can be induced pharmacologically. We report that M2 receptor blockade decreases the frequency and amplitude of locomotor-related activity, whilst reducing its variability. In contrast, M3 receptor blockade destabilizes locomotor-related bursting. Motoneuron recordings from spinal cord slices revealed that activation of M2 receptors induces an outward current, decreases rheobase, reduces the medium afterhyperpolarization, shortens spike duration and decreases synaptic inputs. In contrast, M3 receptor activation elicits an inward current, increases rheobase, extends action potential duration and increases synaptic inputs. Analysis of miniature postsynaptic currents support that M2 and M3 receptors modulate synaptic transmission via different mechanisms. In summary, we demonstrate that M2 and M3 receptors have opposing modulatory actions on locomotor circuit output, likely reflecting contrasting cellular mechanisms of action. Thus, intraspinal cholinergic systems mediate balanced, multimodal control of spinal motor output.

## Introduction

Locomotion is generated by a hierarchy of diverse motor control centres within the nervous system that together program, adapt and execute a plurality of actions. Interestingly, even in the absence of input from other motor control regions, the spinal cord is itself able to produce the basic output needed to generate rhythmic patterns of locomotor activity^1^. Such output is generated by central pattern generator (CPG) circuits, which are embedded within the spinal cord. These CPGs can be modulated by a variety of extrinsic (supraspinal) or intrinsic (intraspinal) systems so that rhythmic patterns of activity can be adjusted to respond to a variety of biomechanical demands^2^.

The cholinergic intraspinal system is an important neuromodulator of locomotor networks. All cholinergic inputs to spinal neurons derive from within the spinal cord^3–5^. Motoneurons are the largest acetylcholine-producing cells of the spinal cord, with their output controlling muscle contraction in the periphery but also activating Renshaw cells centrally, which are responsible for recurrent inhibition of motoneuron pools^6–8^. Groups of cholinergic dorsal interneurons within laminae II-IV are thought to be involved in nociception and inhibition of cutaneous afferent input by modulating the release of GABA and glycine^9–12^. In the ventral horn, besides motoneurons, sources of acetylcholine are restricted to interneurons located in laminae VII and X^3–5,13^. Interneurons expressing the Paired-like homeodomain 2 (Pitx2) transcription factor are the only genetically identified group of spinal cholinergic cells, but they comprise a small portion of the overall cholinergic population in the ventral horn^14,15^. Other subtypes of cholinergic interneurons, such as partition cells that are present in lamina VII close to the central canal^5,13^ and GABAergic and cholinergic cells clustered in lamina X^16^, have been identified based on their anatomical distribution.

Acetylcholine released by spinal neurons can activate both ionotropic (nicotinic) and metabotropic (muscarinic) receptors, which are widely expressed throughout the spinal cord. Nicotinic receptors are important for the activation of Renshaw Cells^7,8,17–20^, modulation of sensory stimuli^12,20–22^ and embryonic development of locomotor networks^23–25^, but are not part of the endogenous cholinergic propriospinal system involved in the modulation of hindlimb locomotor CPG networks^26^. Early evidence indicated that muscarinic receptors can affect motor output^27,28^ with M2 and M3 muscarinic receptors later identified as the subtypes of muscarinic cholinergic receptors that play an active role in the modulation of CPG networks in the mammalian spinal cord^15,29^. Both subtypes of metabotropic receptors are widely distributed in the spinal cord but the role and identity of the spinal neurons expressing M2 and M3 receptors remains unclear^3,30,31^. Regarding M2 receptors, these are known to be expressed on the soma of motoneurons with some clustering juxtaposed with large C-bouton synapses that derive from cholinergic Pitx2^+^ interneurons^15,32–34^. Previous work has failed to detect the expression of M1 and M5 receptors in the spinal cord, whereas M4 receptors seem to be present^31,34^. However, M1, M4 and M5 receptor antagonists have been shown to have no effect on acetylcholine-induced locomotor-like activity in the isolated neonatal spinal cord^26^. This leaves M2 and M3 receptors as the key subtypes involved in the modulation of mammalian spinal motor circuits^11,15,26^.

The roles of M2 and M3 receptors in the modulation of motor output during hindlimb locomotion and their cellular mechanisms of action have not been fully elucidated. Distinct actions of M2 and M3 receptors were demonstrated by experiments performed on neonatal rat spinal cord preparations in which rhythmic locomotor-like activity was induced via administration of the acetylcholinesterase inhibitor edrophonium^26^. In these experiments, antagonism of M2 receptors decreased the amplitude of bursts recorded from ventral roots whereas blockade of M3 receptors lead to a cessation of bursting activity^26^. In the neonatal mouse spinal cord, indirect evidence suggests that M2 receptors can be activated by the output of Pitx2^+^ interneurons, resulting in increased motoneuron firing due to a reduction in the medium afterhyperpolarization (mAHP), following modulation of small conductance calcium-activated potassium channels (SK)^15^. Regarding M3 receptors, these are thought to be involved in long lasting EPSPs recorded from motoneurons after stimulation of commissural cholinergic interneurons in lamina X^13^. Activation of muscarinic receptors has also been associated with modulation of a range of channels responsible for the control of motoneuron excitability such as the hyperpolarization-activated cation current (I_h_), inward rectifying K^+^ current (I_Kir_), resting K^+^ currents and the slowly activating voltage-regulated K^+^ current (M-current)^13,15,35–38^. Given the evidence provided by these previous studies that muscarinic receptors play a range of roles, via differing mechanisms, in the control of locomotor output, we aimed to decipher and directly compare the actions and cellular mechanisms of M2 and M3 receptor-mediated modulation within spinal motor circuits.

In the present study, we report that, due to opposing cellular mechanisms of action, M2 and M3 receptors have contrasting modulatory effects on locomotor control networks of the neonatal mouse spinal cord. M2 receptors increase the variability of locomotor related output, whereas M3 receptors ensure the regularity of locomotor patterns. In addition, M2 and M3 receptors differently modulate motoneuron properties by inducing inward versus outward currents, and differentially altering a range of parameters including input resistance, rheobase, spike half-width and synaptic drive. Thus, we show that acetylcholine derived from spinal sources can have multiple, distinct actions on locomotor networks that are associated with dual control of neuronal excitability by M2 and M3 receptors.

## Materials and Methods

### Tissue preparation

All the procedures performed on animals were conducted in accordance with the UK Animals (Scientific Procedures) Act 1986 and were approved by the University of St Andrews Animal Welfare Ethics Committee. *In vitro* isolated spinal cord preparations were obtained from postnatal day (P)1-P6 C57/BL6 mice, while spinal cord slice preparations were obtained from P2-P12 mice.

Spinal cords were isolated from neonatal mice as previously described^39^. In summary, neonatal mice were euthanized using cervical dislocation, decapitated and eviscerated. The remaining tissue was then pinned ventral side up in a chamber containing “dissecting” artificial cerebrospinal fluid (aCSF) continuously gassed with 95% O_2_ and 5% CO_2_ at a temperature of ~4 °C. Spinal vertebrae were carefully cut and the spinal cord was isolated from cervical to upper sacral segments. To obtain spinal cord slices, both the ventral and dorsal roots were trimmed. Transverse slices were prepared from lumbar segments (300 µm thick) using a vibrating microtome (Leica VT1200). Slices were then transferred to a “recovery” aCSF solution continuously gassed with 95% O_2_ and 5% CO_2_ and at ~34 °C for 45–60 min, before being transferred to a beaker with “recording” aCSF gassed with 95% O_2_ and 5% CO_2_ at room temperature (~20 °C). To perform ventral root recordings, dorsal roots were trimmed and ventral roots from L1-L5 were kept intact. For patch-clamp recordings in intact, *in vitro* isolated spinal cords, small vertical cuts were performed in the ventral meninges on the surface of the preparation next to the ventral root exit points, to allow access to motoneuron pools.

### Electrophysiological recordings

For ventral root recordings, *in vitro* isolated spinal cords were pinned or glued ventral-side up in a recording chamber continuously superfused with recording aCSF. Glass suction electrodes were attached to lumbar ventral roots (L1-L5). N-methyl-D-aspartic acid (NMDA; 5 µM), 5-hydroxytryptamine (5-HT; 10 µM) and dopamine (DA; 50 µM) were added to the aCSF to trigger locomotor-related activity (fictive locomotion). Fictive locomotion was characterized by rhythmic bursts of activity which exhibited alternation between the right and left sides of the spinal cord and between ipsilateral extensor (L5) and flexor (L2) -related ventral roots. Signals were filtered and amplified (band-pass filter 30–3000 Hz, Qjin Design) and then acquired at a frequency of 6 kHz with a Digidata 1440 A A/D board and pClamp software. Custom built amplifiers (Qjin design) allowed acquisition of raw signals with simultaneous online rectification and integration (50-ms time constant).

**Figure 1:**
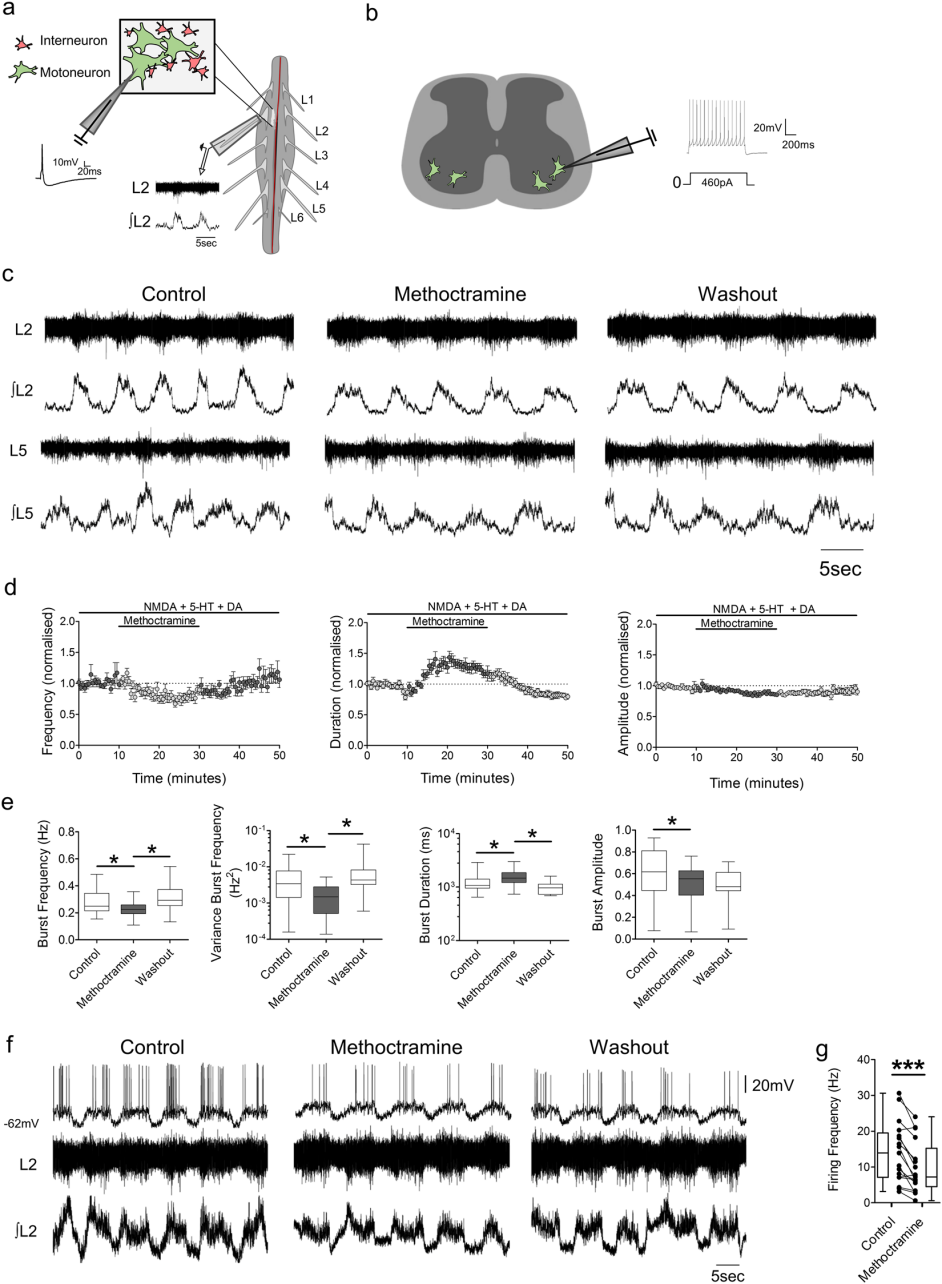
M2 muscarinic receptors modulate active CPG networks and motoneuron firing during drug-induced locomotion. (**a**) Schematics of ventral root and individual motoneuron recordings from intact spinal cords and (**b**) single cell recordings from isolated spinal cord slices. **(c**) Raw (top) and integrated/rectified (bottom) ventral root recordings with **(d)** averaged time course plots and **(e)** box-plots of pooled data illustrating the effects of methoctramine on the frequency, duration and amplitude of drug-induced locomotor output (n = 14). **(f)** Motoneuron firing (top) and simultaneous raw (middle) and integrated/rectified (bottom) ventral root recordings during drug-induced locomotor output. **(g)** Motoneuron firing frequencies plotted for all motoneurons tested showing the effects of methoctramine during drug induced locomotor activity (n = 17). *p < 0.05, ***p < 0.001.

For single cell recordings, *in vitro* isolated spinal cord preparations (Fig. 1a) or spinal cord slices (Fig. 1b) were immersed in a recording chamber continuously superfused with recording aCSF (approximately 1 mL per second). Borosilicated glass microelectrodes (2.5–5 MΩ) were filled with an intracellular solution and used to perform whole-cell patch-clamp recordings from spinal motoneurons. Signals were amplified and filtered (4-kHz low-pass Bessel filter) with a MultiClamp 700B amplifier (Molecular Devices, Sunnyvale, CA, USA) and acquired at ≥10 kHz using a Digidata 1440 A A/D board and pClamp software (version 10.6, Molecular Devices, Sunnyvale, CA, USA). A gigaohm seal (≥2 GΩ) was obtained, prior to the establishment of whole-cell configuration in which the neuron was typically held at −60 mV when in voltage-clamp mode. Neurons with an RMP between −45 and −80 mV with an access resistance ≤20 MΩ were used for recordings. Acquisition of spontaneous postsynaptic currents (PSCs), miniature postsynaptic currents (mPSCs), drug-induced currents and measurements of input resistance (by applying voltage steps; 2.5 mV steps from −75 to −52.5 mV) were performed in voltage-clamp mode (V_hold_ −60 mV). Firing output was measured in current-clamp mode either using ‘gap-free’ acquisition for spontaneous firing during fictive locomotion, by applying 10 ms supramaximal pulses to evoke single action potentials, or by injecting 1 s square current pulses (starting at 10 pA with 50 pA increments). Cells with overshooting spikes ≥60 mV were used for analysis. To facilitate comparisons, a bias current was applied in all current-clamp protocols to keep neurons at the same resting potential (approximately −60 mV).

### Drugs and solutions

The dissecting aCSF contained (in mM): 25 NaCl, 188 sucrose, 1.9 KCl, 1.2 NaH_2_PO_4_, 10 MgSO_4_, 1 CaCl_2_, 26 NaHCO_3_, 25 glucose and 1.5 kynurenic acid. The recovery solution contained (in mM): 119 NaCl, 1.9 KCl, 1.2 NaH_2_PO_4_, 10 MgSO_4_, 1 CaCl_2_, 26 NaHCO_3_, 20 glucose and 1.5 kynurenic acid. The recording aCSF contained (in mM): 127 NaCl, 3 KCl, 1.25 NaH_2_PO_4_, 1 MgCl_2_, 2 CaCl_2_, 26 NaHCO_3_, 10 glucose. The intracellular solution for patch-clamp recordings was made of (in mM): 140 KMeSO_4_, 10 NaCl, 1 CaCl_2_, 10 HEPES, 1 EGTA, 3 Mg-ATP and 0.4 GTP-Na_2_ (pH 7.2–7.3, adjusted with KOH). Muscarine, 4-DAMP, methoctramine, NMDA, DA and 5-HT were supplied by Sigma-Aldrich®; tetrodotoxin (TTX) by Bio-Techne®. All drugs were dissolved in H_2_O except for 4-DAMP which was dissolved in DMSO to a concentration that did not exceed 0.1% (vol/vol) in working solutions.

### Data analysis

Single cell recordings were analyzed with Clampfit software (Molecular Devices, Sunnyvale, CA, USA) or, for PSCs and mPSCs, using Mini-Analysis software (Synaptosoft Inc, Decatur, GA). For quantification and statistical analyses of synaptic inputs, the last 1–2 minutes or at least 80–100 events from control, drug and washout were used. Changes in holding current were calculated as the difference between the current value immediately before drug perfusion and the maximum change in current induced by the drug. Current-voltage relationships were constructed for drug-induced currents by subtracting the voltage step values from control from those obtained after drug perfusion. Linear regression of these values was used to calculate the reversal potential of drug-induced currents (X-intercept when Y = 0.0). For analysis of rheobase, the current values of the first steps eliciting neuronal firing during control and drug were compared. For maximum firing output, the current steps eliciting the highest firing frequency during control and drug conditions were compared. Single action potential mAHP amplitude was considered as the difference between the resting voltage before injecting the 10 ms-long depolarizing current and the peak value of the mAHP (averaged across 15 traces). For the analysis of action potential half-width and rise-time, individual spikes in each condition were used. For each spike a differential function (dV/dt) was calculated and the point where dV/dt reached 10 mV/ms was set as the action potential threshold used for the estimation of half-width and rise time. The values obtained from four or more action potentials were then averaged and used for statistical comparisons. Subthreshold and firing properties of recorded motoneurons are summarized in Table 1.

**Table 1:** Properties of motoneurons recorded.

Input resistance (MΩ)	Capacitance (pF)	Resting membrane potential (mV)	Rheobase (pA)	mAHP amplitude (mV)	Maximum firing (Hz)	Half-width (ms)	Rise time (ms)
66 ± 28 (n = 82)	134 ± 41 (n = 82)	−60 ± 8 (n = 82)	337 ± 303 (n = 47)	−5.3 ± 3.9 (n = 48)	34 ± 21 (n = 40)	1.2 ± 0.3 (n = 51)	0.6 ± 0.2 (n = 51)

Ventral root bursts were analyzed offline using DataView software (courtesy of Dr W. J. Heitler, University of St Andrews). Bursts were identified and their frequency and duration measured from the integrated/rectified traces. Following identification of bursts from integrated/rectified traces, burst amplitude was calculated from the respective segment of raw trace and measured as a non-calibrated component reported in arbitrary units. Burst regularity was measured as the variance of burst frequency. Data were averaged in 0.5-min time bins and normalized to a 10 min pre-control period to construct time course plots. Statistical comparisons were made on raw data averaged over 5-min periods in each condition.

In the text, data are reported as mean ± standard deviation. In the figures, values are represented as box plots displaying the distribution of data as the minimum, first quartile, median, third quartile, and maximum value for each dataset. For patch-clamp experiments “n” corresponds to the number of cells, whereas for ventral root recordings “n” corresponds to the number of *in vitro* isolated spinal cord preparations. GraphPad Prism Software version 5.1 (GraphPad Software, La Jolla, CA) was used to conduct statistical analysis and plot data, with asterisks in the figures indicating statistical significance (p < 0.05). D’Agostino-Pearson tests were used to assess normality. Repeated measures ANOVA followed by Tukey post-hoc test or Friedman’s test with Dunns post-test were used to test for statistically significant differences when comparing more than 2 conditions. Paired *t*-tests or Wilcoxon matched pairs tests were used for comparing differences in means between 2 paired conditions; drug and control. Unpaired t-tests were used to calculate differences in capacitance between different groups of motoneurons.

## Results

### Blockade of M2 muscarinic receptors effects the regularity, decreases the frequency and reduces the amplitude of drug-induced locomotor output

We first investigated the modulatory actions of M2 muscarinic receptors within spinal locomotor networks by applying the M2 receptor antagonist methoctramine while recording drug-evoked (NMDA, 5 µM; 5-HT, 10 µM; and DA, 50 µM), stable, fictive locomotion via suction electrodes attached to the lumbar ventral roots (Fig. 1c–e). Methoctramine was applied at a concentration of 10 µM, which has previously been shown to effect locomotor-like rhythms recorded from *in vitro* isolated spinal cord preparations^26,40^. Blockade of M2 receptors decreased burst frequency (−14.7 ± 22.6%, p < 0.001, Friedman’s test, n = 14) and reduced the variability in burst frequency (−56.3 ± 21.4%, p < 0.001, Friedman’s test, n = 14). Application of methoctramine during fictive locomotion also increased the duration of the ventral root bursts (29.1 ± 22.1%, p < 0.001, Friedman’s test, n = 14) and reduced their amplitude (−12.3 ± 9.6%, p = 0.0025, Friedman’s test, n = 14). To investigate whether M2 receptor blockade during drug-induced bursting impacted motoneuron firing rates, we performed whole-cell patch-clamp recordings from single motoneurons that were rhythmically active within *in vitro* isolated spinal cord preparations during fictive locomotion. As depicted in Fig. 1f,g, methoctramine significantly decreased motoneuron firing frequency during locomotor episodes (t(16) = 5.01, p < 0.001, n = 17, paired t-test). These data indicate that activation of M2 receptors by acetylcholine derived from within the spinal cord modulates the frequency, duration and amplitude of locomotor-related output. Changes in locomotor burst amplitude probably reflect modulation of motoneuron firing rates.

### Blockade of M3 muscarinic receptors disrupts drug-induced locomotor output

**Figure 2:**
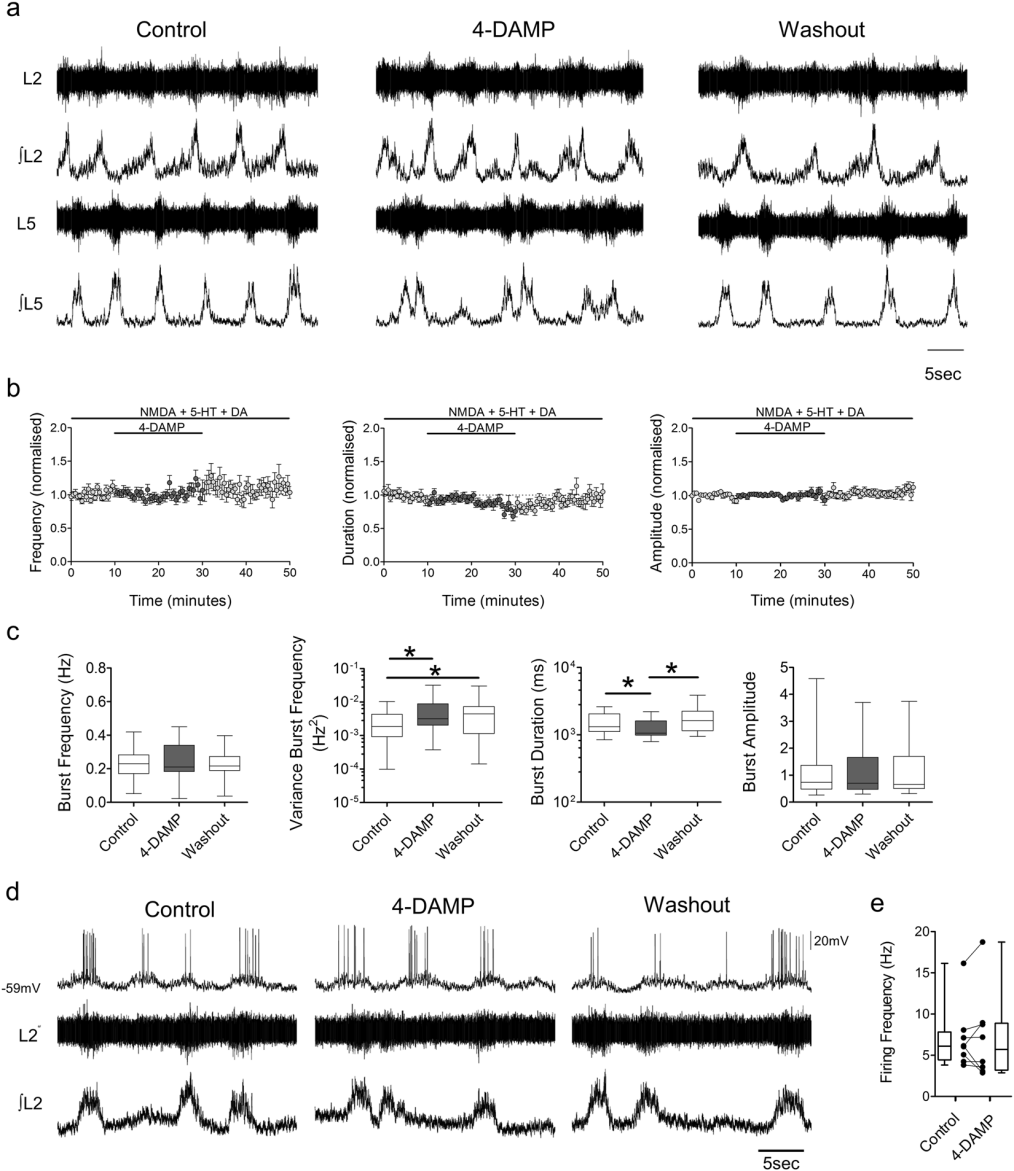
M3 receptors modulate the stability of the locomotor rhythm during drug-induced activity. **(a)** Raw (top) and integrated/rectified (bottom) ventral root recordings with **(b)** averaged time course plots and **(c)** box-plots of pooled data showing the effects of 4-DAMP on the frequency, duration and amplitude of drug-induced locomotor output (n = 22). **(d)** Motoneuron firing (top), and simultaneous raw (middle) and integrated/rectified (bottom) ventral root recordings during drug-induced locomotor output. **(e)** Motoneuron firing frequencies plotted to illustrate the effects of 4-DAMP during drug induced locomotor activity (n = 8). *p < 0.05.

To assess the modulatory roles of M3 receptors within spinal locomotor circuits, we perfused the M3 receptor antagonist 4-DAMP during drug-induced fictive locomotion (Fig. 2a–c). 4-DAMP was applied at a concentration of 2 µM, within the range that has previously been used to block M3 receptor-related locomotor activity in the spinal cord^13,26^. Application of 4-DAMP did not significantly affect burst frequency (p = 0.834, Friedman’s test, n = 22) but did lead to a greater variance in burst frequency (159.5 ± 203.2%, p < 0.001, Friedman’s test, n = 22). Blockade of M3 receptors reduced burst duration (−14.6 ± 14.5%, p = 0.003, Friedman’s test, n = 22) but had no significant effect on burst amplitude (p = 0.483, Friedman’s test, n = 22). Recordings from single motoneurons during fictive locomotion, showed that perfusion of 4-DAMP did not affect motoneuron firing frequency (Fig. 2d,e, t(7) = 0.128, p = 0.905, n = 8, paired t-test). These results indicate that activation of M3 receptors by spinally-derived acetylcholine is important for stabilizing rhythmic, locomotor-related output, but has little effect on the intensity of motor output.

### M2 receptor activation elicits an outward current while M3 receptor activation induces an inward current in motoneurons

Having established the modulatory effects of M2 and M3 receptors on spinal locomotor network output, we next investigated the cellular mechanisms of action of muscarinic receptors by applying muscarine alone or in combination with M2 or M3 receptor antagonists during whole-cell patch-clamp recordings of motoneurons in spinal cord slices.

**Figure 3:**
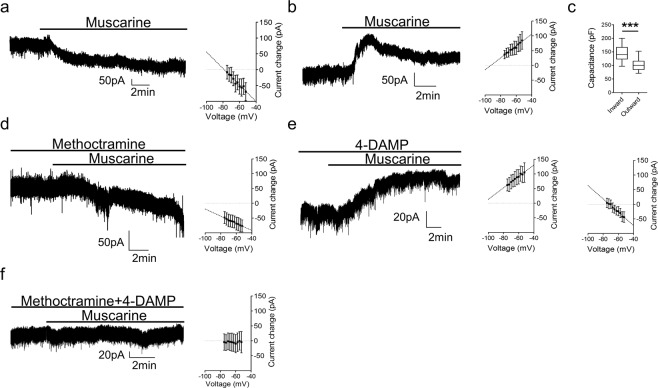
Muscarine preferentially induces a M2 receptor-mediated outward current in smaller motoneurons and a M3 receptor-dependent inward current in larger motoneurons. **(a)** Voltage-clamp recordings illustrating an inward current and **(b)** outward current elicited by muscarine with respective I-V plots depicting an increase (n = 9) or decrease (n = 6) in input resistance. **(c)** Plot of average capacitance values for motoneurons exhibiting inward versus outward currents in response to muscarine. (**d**) Illustrative trace of the inward current elicited by muscarine in the presence of methoctramine with respective I-V plot (n = 8). (**e**) Representative trace of the outward current induced by muscarine co-applied with 4-DAMP and I-V plots for motoneurons that showed an increase (left, n = 6) or decrease in input resistance (right, n = 9). (**f**) Muscarine in the presence of both M2 and M3 receptor antagonists did not affect membrane current (n = 10) or input resistance (n = 7). All recordings were performed at a holding voltage of −60 mV. ***p < 0.001.

To explore changes in neuronal excitability mediated by muscarinic receptors, we first evaluated the effects of muscarine (10 µM) application on the subthreshold properties of motoneurons measured in voltage-clamp mode. In the presence of muscarine, two different responses were observed. In one subset of motoneurons, muscarine induced an inward current (Fig. 3a, −57 ± 36 pA n = 26), which was associated with an increase in input resistance (control: 93 ± 64 MΩ, muscarine: 120 ± 80 MΩ, reversal −78.43 mV, n = 9). In another group of motoneurons, muscarine elicited an outward current (Fig. 3b, 41 ± 38 pA; n = 11) which was associated with a decrease in input resistance (control: 139 ± 49 MΩ, muscarine: 120 ± 46 MΩ, reversal −93 mV, n = 6). Motoneurons in which an inward current was observed were larger, as indicated by whole-cell capacitance values, than those in which an outward current was induced (Fig. 3c, t(35) = 5.025, p < 0.001, unpaired t-test).

To understand which receptor subtypes were responsible for the different muscarine-induced currents, we co-perfused muscarine with M2 or M3 receptor antagonists. In the presence of the M2 receptor antagonist methoctramine, muscarine only elicited an inward current (Fig. 3d, −52 ± 28 pA, n = 19) which was associated with an increase in input resistance (methoctramine: 103 ± 37 MΩ, methoctramine and muscarine: 118 ± 48 MΩ, reversal −116 mV, n = 8). In contrast, when M3 receptors were blocked with 4-DAMP, muscarine only elicited an outward current (Fig. 3e, 55 ± 37 pA, n = 18). These outward currents were associated with a decrease in input resistance in some motoneurons (4-DAMP: 117 ± 55 MΩ, 4-DAMP and muscarine: 86 ± 49 MΩ, reversal −107 mV, n = 6) and an increase in input resistance in others (4-DAMP: 67 ± 15 MΩ, 4-DAMP and muscarine: 81 ± 20 MΩ, reversal −72 mV, n = 9). There was no significant difference in capacitance between motoneurons that exhibited an outward current with decreased versus increased input resistance (t(14) = 0.347, p = 0.734). The same effects of muscarine in the presence of the antagonists were observed when co-applied with TTX (0.5 µM) (Supplementary Fig. 1), indicating that changes in subthreshold properties occur due to activation of postsynaptic muscarinic receptors on motoneurons. Finally, to test for involvement of any other subtypes of muscarinic receptors, muscarine was applied in the presence of both methoctramine and 4-DAMP while recording from motoneurons. As seen in Fig. 3f, in the presence of methoctramine and 4-DAMP, muscarine had no effect on holding current (1 ± 10 pA, n = 10) or input resistance (4-DAMP and methoctramine: 97 ± 64 MΩ, 4-DAMP, methoctramine and muscarine: 95 ± 47 MΩ, n = 7). These data demonstrate that muscarine acts via M2 and M3 receptors on motoneurons and that these two receptor subtypes have opposing actions.

### M2 and M3 muscarinic receptors differently affect motoneuron rheobase and are both involved in muscarine-dependent increases in maximum firing

**Figure 4:**
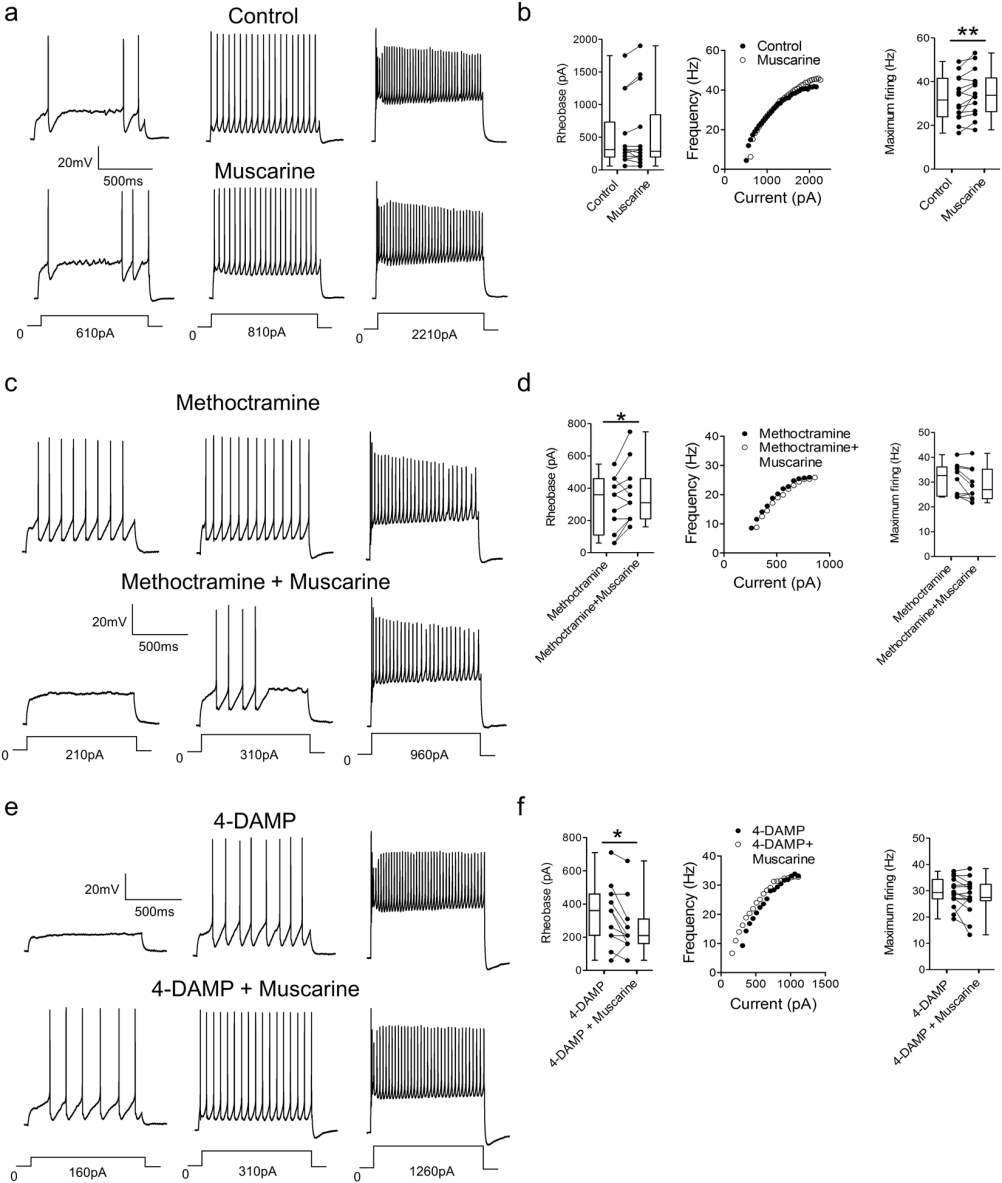
M2 and M3 receptors differently affect rheobase and both modulate motoneuron firing rates. **(a)** Current-clamp recordings showing the effects of muscarine on motoneuron firing in response to current injection. **(b)** Graphs showing the effects of muscarine on rheobase (n = 14), an illustrative f-I relationship and maximum firing frequencies (n = 14). **(c)** Motoneuron firing in response to current injection illustrating the effects of muscarine in the presence of methoctramine. **(d)** Graphs showing the effects of muscarine, in the presence of methoctramine, on rheobase (n = 11), an illustrative f-I relationship and maximum firing (n = 10). **(d)** Motoneuron firing in response to current injection illustrating the effects of muscarine in the presence of 4-DAMP. **(f)** Graphs showing the effects of muscarine, in the presence of 4-DAMP, on rheobase (n = 11), an illustrative f-I plot and maximum firing (n = 15). *p < 0.05.

To investigate the modulatory effects of muscarinic receptor activation on motoneuron output, muscarine was perfused while trains of action potentials were elicited via current injection (1 s duration) in current-clamp mode. Repetitive firing was induced using a series of current steps of increasing magnitude. Comparisons of repetitive firing elicited in control conditions and in the presence of muscarine revealed that muscarinic receptor activation did not change rheobase (Fig. 4a,b, p = 0.230, n = 14, Wilcoxon matched pairs test) but significantly increased motoneuron maximum firing rate (t(13) = 3.022, p = 0.009, n = 14, paired t-test). To address the role of M3 receptors in the modulation motoneuron firing properties, motoneuron output was studied during perfusion of muscarine in the presence of the M2 receptor antagonist methoctramine. As illustrated in Fig. 4c,d, muscarine, when co-applied with methoctramine, increased rheobase (t(10) = 2.353, p = 0.040, n = 11, paired t-test) but did not affect maximum firing rates of motoneurons (t(9) = 2.197, p = 0.056, n = 10, paired t-test). To reveal the potential impact of M2 receptor activation on motoneuron firing output, muscarine was next perfused with 4-DAMP. As illustrated in Fig. 4e,f, muscarine, applied in the presence of 4-DAMP, decreased motoneuron rheobase (p = 0.042, n = 11, Wilcoxon matched pairs test) but did not affect maximum motoneuron firing rates (t(14) = 1.463, p = 0.165, n = 15, paired t-test). These data again demonstrate opposing modulatory actions of M2 and M3 receptors, this time with respect to regulation of the amount of stimulation required to induce repetitive firing in motoneurons. Data also indicate that both receptor subtypes contribute to muscarine-dependent increases in maximum motoneuron firing rates.

### M2 receptors modulate the medium afterhyperpolarization and decrease spike half-width while M3 receptors increase action potential duration

Given previous reports that muscarinic receptor activation affects motoneuron firing through the modulation of the mAHP^15^, we next investigated the effects of M2 and M3 receptor activation on the mAHP in motoneurons. Short current steps (10 ms) were used to elicit single action potentials followed by clear mAHPs. The magnitude of mAHPs as well as action potential half-width and rise-time were measured during activation of muscarinic receptors.

**Figure 5:**
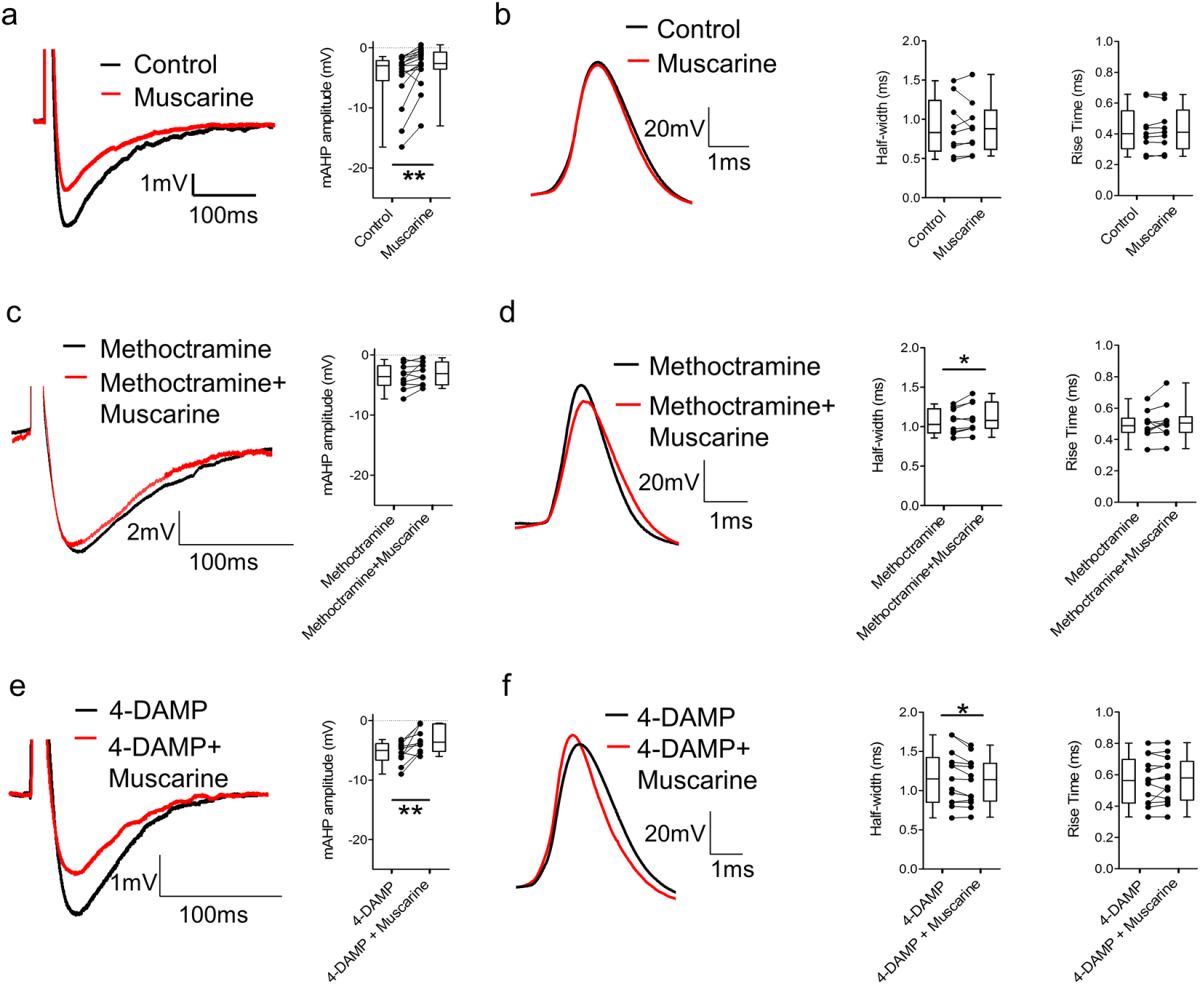
M2 receptor activation modulates the mAHP and decreases spike half-width while M3 receptor activation increases action potential duration. **(a)** Truncated single action potentials showing the effects of muscarine on the mAHP (n = 17). **(b)** Superimposed action potentials recorded from a motoneuron before and after muscarine with plots of average values of action potential half-width and rise-time in each condition (n = 9). **(c)** Truncated single action potentials illustrating the effect of muscarine co-applied with methoctramine on the mAHP (n = 10). **(d)** Representative traces of action potentials in the presence of methoctramine or muscarine and methoctramine, with plots of average half-width and rise rime (n = 10). **(e)** Truncated single action potentials illustrating the effect of muscarine co-applied with 4-DAMP on the mAHP (n = 10). **(f)** Representative traces of action potentials in the presence of 4-DAMP or muscarine and 4-DAMP, with plots of average half-width and rise-time (n = 13). *p < 0.05, **p < 0.01.

Application of muscarine decreased mAHP amplitude (Fig. 5a, p = 0.002, n = 17) but did not change single action potential half-width (Fig. 5b, t(8) = 0.041, p = 0.968, n = 9) or rise-time (t(8) = 0.660, p = 0.528, n = 9). In the presence of the M2 antagonist methoctramine, application of muscarine had no effect on mAHP amplitude (Fig. 5c, t(9) = 1.908, p = 0.089, n = 10) but we detected an increase in spike half-width (Fig. 5d, t(9) = 2.829, p = 0.022, n = 10) without any significant effect on rise-time (t(9) = 1.334, p = 0.215, n = 10). When co-applied with the M3 receptor blocker 4-DAMP, muscarine reduced the mAHP amplitude (Fig. 5e, t(9) = 3.793, p = 0.004, n = 10) and decreased action potential half-width (Fig. 5f, t(12) = 2.952, p = 0.012, n = 13) without affecting rise-time (t(12) = 0.873, p = 0.400, n = 13). Results from single action potential analysis indicate that M2 receptors are involved in the modulation of the mAHP, as previously reported^15^. In addition, we reveal further opposing modulatory actions of M2 and M3 receptors, specifically on action potential kinetics.

### M2 and M3 receptors differently modulate synaptic inputs to motoneurons

**Figure 6:**
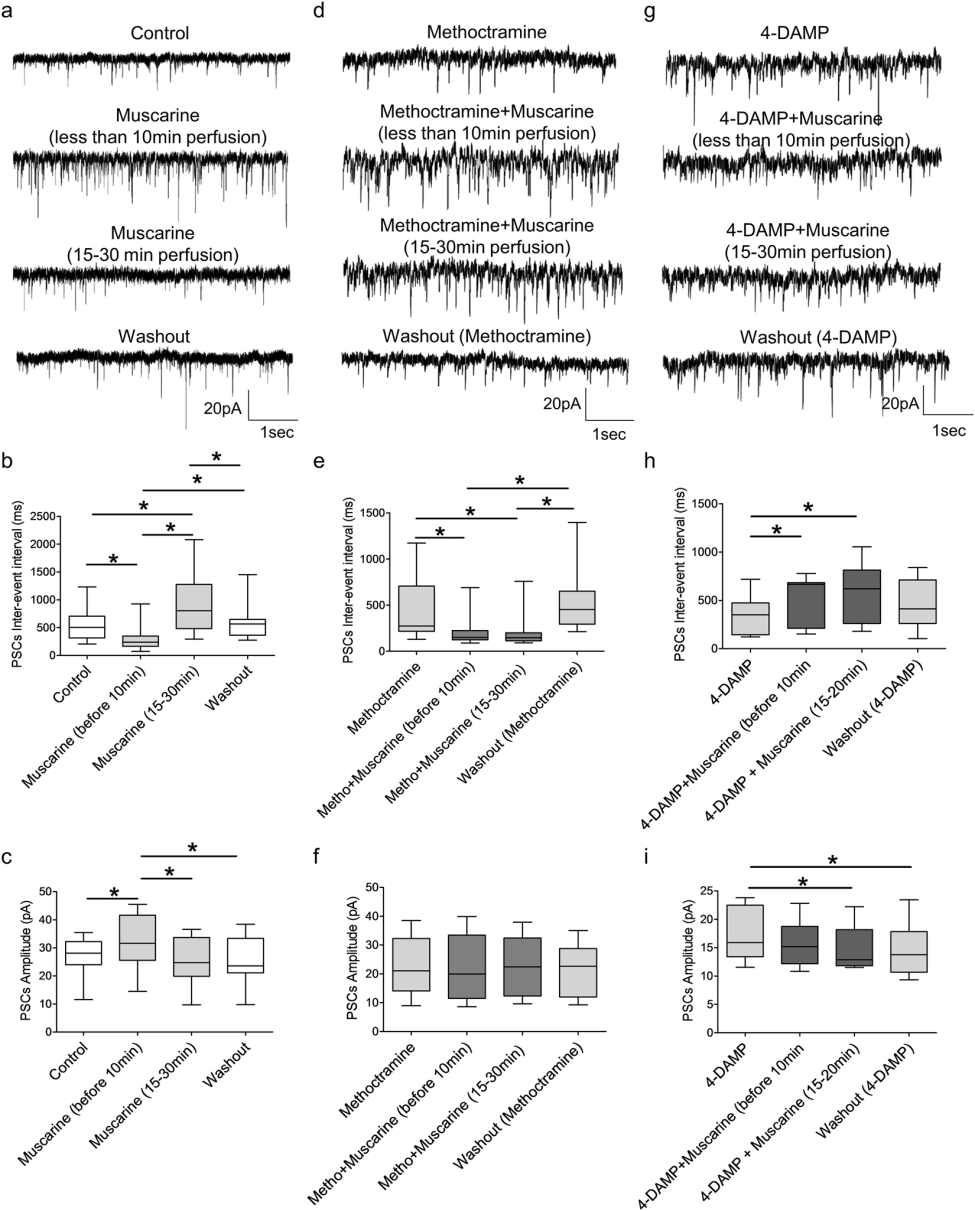
Muscarine has a biphasic effect on the synaptic drive to motoneurons. **(a)** Representative voltage-clamp traces of PSCs recorded before, during and after muscarine application. Box-plots showing the time-dependent effects of muscarine on PSC inter-event interval **(b)** and PSC amplitude **(c)** (n = 12). **(d)** Example traces showing the effects of muscarine on PSCs when applied in the presence of methoctramine. Box-plots showing the effects of muscarine co-applied with methoctramine on PSC inter-event interval **(e)** and PSC amplitude **(f)** (n = 12). **(g)** Example traces showing the effects of muscarine on PSCs when co-applied with 4-DAMP. Box-plots showing the effects of muscarine co-applied with 4-DAMP on PSC inter-event interval **(h)** and PSC amplitude **(i)** (n = 10). All recordings were performed at a holding potential of −60 mV. *p < 0.05.

Finally, we assessed the potential modulatory effects of M2 and M3 receptor activation on synaptic inputs to motoneurons. Mixed PSCs were recorded from motoneurons while muscarinic receptors were either activated or inhibited. The general agonist muscarine had an initial excitatory (first 10 minutes of perfusion) followed by a delayed inhibitory effect on the frequency of synaptic events. This was revealed by an early decrease, followed by a later increase in inter-event interval (Fig. 6a–c, p < 0.001, repeated measures ANOVA, n = 12). We also observed a transient increase (initial 10 minutes) in the amplitude of PSCs recorded from motoneurons (p < 0.001, repeated measures ANOVA, n = 12). The modulatory roles of M3 receptors on synaptic drive were first assessed by applying muscarine in the presence of the M2 antagonist to isolate actions mediated by M3 receptors. When co-perfused with methoctramine, muscarine decreased PSC inter-event interval (Fig. 6d–f, p < 0.001, Friedman’s test, n = 12) but had no effect on PSC amplitude (p = 0.631, repeated measures ANOVA, n = 12). Next, the M3 receptor blocker 4-DAMP was co-perfused with muscarine to assess the modulatory actions of M2 muscarinic receptor activation on synaptic inputs to motoneurons. Muscarine in the presence of 4-DAMP increased PSC inter-event interval (Fig. 6g–i, p = 0.002, repeated measures ANOVA, n = 10) and decreased PSC amplitude (p = 0.007, repeated measures ANOVA, n = 10). These results indicate that muscarinic receptor activation has two distinct, time-dependent modulatory actions on synaptic inputs to motoneurons: an initial increase mediated by M3 receptors followed by a subsequent decrease in synaptic drive that is dependent on M2 receptor activation.

**Figure 7:**
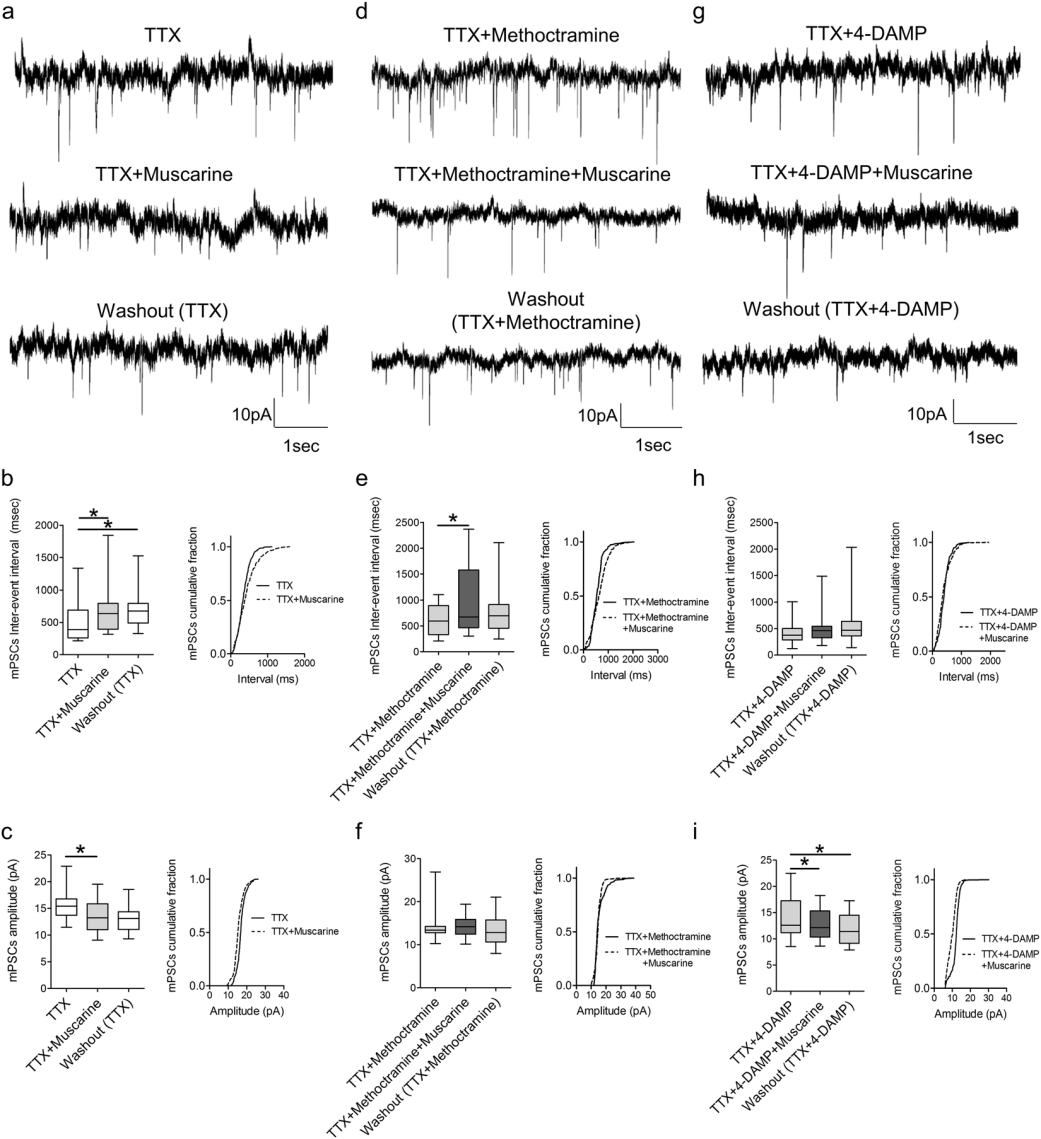
Muscarine modulates both the amplitude and frequency of miniature postsynaptic currents through M2 and M3 receptors. **(a)** Voltage-clamp recordings of mPSCs (with 0.5 μM TTX) before, during and after application of muscarine. Box plots and cumulative frequency plots of mPSC inter-event interval **(b)** and mPSC amplitude **(c)** showing the effects of muscarine on spontaneous activity (n = 15). **(d)** Example traces of mPSCS recorded in the presence of muscarine and methoctramine. Box plots and cumulative frequency plots of mPSC inter-event interval **(e)** and mPSC amplitude **(f)** showing the effects of muscarine when co-applied with methoctramine (n = 16). (**g)** Representative traces of mPSCs recorded in the presence of 4-DAMP and muscarine. Box plots and cumulative frequency plots of mPSC inter-event interval **(h)** and mPSC amplitude **(i)** showing the effects of muscarine when co-applied with 4-DAMP (n=16). All recordings were performed at a holding potential of −60 mV. *p < 0.05.

Muscarinic receptor-dependent modulation of synaptic activity could reflect reduced output from presynaptic neurons or direct modulation of synaptic transmission via pre- and/or postsynaptic mechanisms. To address these possibilities, action potential-mediated synaptic inputs were blocked with TTX (0.5 μM), and the remaining miniature (m)PSCs, which result from spontaneous vesicular release of transmitter, were analysed during perfusion of muscarine alone or in the presence of the M2 or M3 receptor antagonists. As illustrated in Fig. 7a–c, muscarine increased the inter-event interval (p = 0.002, Friedman’s test, n = 15) and decreased the amplitude (p < 0.001, repeated measures ANOVA, n = 15) of recorded mPSCs. In the presence of the M2 receptor blocker, methoctramine, muscarine increased the inter-event interval (Fig. 7d–f, p = 0.017, repeated measures ANOVA, n = 16) but did not change the amplitude (p = 0.494, Friedman’s test, n = 16) of mPSCs. Perfusion of muscarine during blockade of M3 receptors with 4-DAMP revealed no change in mPSCs inter-event interval (Fig. 7g–i, p = 0.175, Friedman’s test, n = 16) but a decreased in their amplitude (p < 0.001, repeated measures ANOVA, n = 16). These data indicate differential mechanisms of modulation of synaptic activity by muscarinic receptors with M2 receptors potentially acting via postsynaptic and M3 receptors via presynaptic mechanisms.

## Discussion

In the present study we have demonstrated that acetylcholine, derived from sources within the mouse spinal cord, modulates spinal locomotor control circuitry via M2 and M3 muscarinic receptors. Interestingly, we reveal differential effects of M2 and M3 receptor activation on locomotor-related output, which reflect opposing cellular mechanisms of action. Our findings build on previous evidence of opposing actions of neuromodulators within CPG circuits, which have been hypothesized to allow state-dependent flexibility but also constrain CPG activity within an optimal operational range (see^41^ for review).

Blockade of M2 receptors during stable, left-right and flexor-extensor alternating, rhythmic locomotor-related activity revealed an endogenous role for these receptors in the modulation of CPG neurons important in the regulation of locomotor frequency and locomotor burst duration. Previous studies utilising the rat spinal cord have also reported changes in the frequency of locomotor-related activity due to activation of M2 receptors^26,42^. However, while we observed a decrease in the frequency of locomotor-related output upon application of the M2 receptor antagonist methoctramine, previous studies reported an increase in frequency with methoctramine when also using the acetylcholinesterase inhibitor edrophonium to increase cholinergic signaling^26,42^. These differences may relate to the species used or varying concentrations of acetylcholine due to the use of acetylcholinesterase inhibitors. Nevertheless, our study provides further evidence that endogenous acetylcholine acting via M2 receptors, regulates the activity of CPG neurons involved in rhythm generation. In addition to this, we also show methoctramine-dependent effects on the amplitude of the locomotor bursts revealing that, as previously suggested^15,26,42^, M2 receptors are important for enhancing the strength of motor output.

M3 receptor blockade destabilized the fictive locomotor rhythm without changing burst amplitude, indicating that the most prominent effects of M3 receptor activation by spinally-derived acetylcholine likely involve modulation of spinal interneurons involved in rhythm and pattern generation. In the rat parafacial respiratory group^43^ and the preBötzinger complex^44,45^ M3 receptors are involved in the excitation of neurons that are crucial for the generation of adequate frequencies of respiratory patterns, while in the lumbar spinal cord of rats M3 receptor activation can elicit well-defined locomotor-like bursts of activity^26^. Given the structural and functional similarities between brainstem and spinal CPGs^46,47^, it may follow that M3 receptor-mediated modulation of the mouse locomotor circuitry is important for ensuring adequate rhythmic patterns of activity. M3 receptors are present in the ventral horn but their specific distribution amongst different types of interneurons is still unclear^31,34^. M3 receptors are thought to inhibit the M-current, whose activation is known to reduce cell excitability^13,48–50^. Thus, blockade of M3 receptors within the spinal locomotor CPG using 4-DAMP could prevent cholinergic downregulation of the M-current, which could in turn decrease the activity of the CPG kernel involved in maintaining burst regularity.

The exact origin of the cholinergic inputs responsible for modulation of CPG interneurons is unclear. A variety of different populations of premotor acetylcholine-releasing interneurons are known to project intersegmentally, including cholinergic neurons from sacral segments which project to the lumbar region^51,52^. Acetylcholine-releasing interneurons are also known to preferentially cluster around the ventromedial region of the spinal cord^3,5,53–57^. From these, cholinergic Pitx2^+^ interneurons have been genetically identified^14^. A small percentage of Ia inhibitory interneurons receives inputs from cholinergic Pitx2^+^ interneurons^7,58,59^ while V2a interneurons have sparse cholinergic innervation^14^. However, these classes of CPG neurons are not thought to shape the duration or frequency of locomotor bursts but are instead involved in extensor-flexor and left-right reciprocal alternation, respectively^60,61^. The decrease in ventral root amplitude and motoneuron spiking during bursting, which we observed during muscarine receptor blockade, could be attributed to blockade of C-bouton-derived inputs to motoneurons that have been suggested to increase motor output during locomotion through the activation of M2 receptors^14,15^.

To examine the cellular mechanisms of M2 and M3 receptor modulation of locomotor networks, we first explored the effects of muscarinic receptor activation on subthreshold properties of motoneurons in spinal cord slices. We observed a M2 receptor-mediated outward current that was most likely due to the opening of leak K^+^ channels, as indicated by a decrease in input resistance and a reversal potential close to the equilibrium potential for K^+^ in our solutions (−98 mV). Similar results have been reported in salamander motoneurons where muscarine induced an outward current and decreased input resistance by affecting I_KIR_^38^ and in sympathetic preganglionic neurons where M2 receptor-dependent outward currents have been attributed to modulation of K^+^-conducting channels^62^. Interestingly, we observed a M2-receptor dependent increase in input resistance in a subset of motoneurons. In our experimental setup muscarine interacts with M2 receptors in a diffuse manner, activating both synaptic and extra synaptic receptors and we are therefore measuring the overall effect of what may be multiple, normally separate, pathways by which M2 receptor activation affects motoneuron properties. Thus, differences in the net effect of M2 receptor activation could reflect the relative strength of different M2 receptor-mediated pathways in different subtypes of motoneurons. Contrasting with M2 receptor activation, M3 receptors elicited an inward current and increased input resistance in motoneurons. Activation of spinal cholinergic interneurons has been shown to depolarize motoneurons through M3 receptors, presumably via inactivation of the M-current^13,48–50^. Blocking the M-current can depolarize spinal motoneurons and increase their input resistance^48,49^ suggesting that the M3 receptor-dependent inward current and increased resistance we observed could result from regulation of M-channels.

When investigating the effects of muscarinic receptor activation on motoneuron output, we observed a muscarine-induced increase in maximum firing that was blocked by both M2 and M3 receptor antagonists. Previous, indirect evidence has suggested that muscarine increases motoneuron output through the activation of M2 receptors located at C-bouton synapses^15,63^, which could explain our findings that methoctramine application reduces both motoneuron firing and the amplitude of locomotor-related activity recorded from ventral roots. As discussed above, M3 receptors might downregulate a tonically active M-current whose activity reduces the excitability of motoneurons and limits maximum firing frequency^13,48–50^. In fact, blockade of the active M-current has been shown to decrease rheobase and firing in spinal motoneurons^48^ which is similar to our observations when 4-DAMP was applied alone to motoneurons (Supplementary Fig. 2). Therefore, considering that the M3 receptor antagonist itself decreased motoneuron firing output by reducing motoneuron excitability, M3 receptor blockade might also prevent muscarine-induced changes in maximum firing frequency. Irrespective of the exact mechanisms, our data show that both M2 and M3 receptors can modulate motoneuron firing output.

To further explore the mechanisms by which M2 and M3 receptor activation modulates the output of motoneurons we evoked single action potentials and analyzed the mAHP and spike duration. M3 receptor activation did not change mAHP amplitude but increased spike half-width, a feature previously observed in basket cells upon M3 receptor activation^64^. The M-current is not thought to affect spike half-width in motoneurons^48^, indicating that the observed M3 receptor-dependent effects on spike parameters might involve other, as yet unidentified, targets. M2 receptor activation reduced mAHP amplitude and shortened spike duration by reducing the half-width of action potentials without significantly affecting rise time. M2 receptors juxtaposed to C-boutons are clustered next to SK channels which are known to underlie the mAHP of motoneurons^15^. Kv2.1 channels are also located next to these postsynaptic clusters of M2 receptors^34^ and changes in Kv2.1 currents can limit motoneuron firing output by reducing spike half-width^65^. Thus, the reduction in mAHP amplitude and action potential duration might result from activation of M2 receptors opposite C-boutons followed by subsequent regulation of SK and Kv2.1 channels.

We also explored the muscarinic modulation of synaptic inputs to motoneurons. We found that M2 receptor activation decreased, while M3 receptor activation increased, the frequency of PSCs to motoneurons. This could reflect direct modulation of synaptic transmission, given that M2 receptor activation decreases glutamatergic transmission from primary sensory afferents to dorsal neurons^66^ and decreases evoked AMPA-mediated synaptic currents recorded from mouse motoneurons after stimulation of the spinal cord dorsolateral funiculus^67^, while blockade of M3 receptors was shown to affect motoneuron potentials in response to stimulation of spinal commissural interneurons^13^. However, given significant expression of M2 receptors in the mid-lumbar spinal cord of rats^68^, and the demonstration that M3 receptors excite lumbar motor networks resulting in the generation of bursts of ventral root activity in the rat^26^, activation of M2 and M3 receptors could also differentially alter the activity of interneurons resulting in indirect changes in synaptic drive to motoneurons. In support of M2 receptor-mediated modulation of synaptic inputs to motoneurons, we observed a reduction in the amplitude of mPSCs recorded from motoneurons in the presence of TTX. These data support postsynaptic modulation triggered by the activation of M2 receptors located on the motoneuron soma, potentially those clustered opposite C-boutons. We also found that the frequency of mPSCs was reduced following M3 receptor activation, supporting the existence of M3 receptor-mediated presynaptic modulation of synaptic transmission.

**Figure 8:**
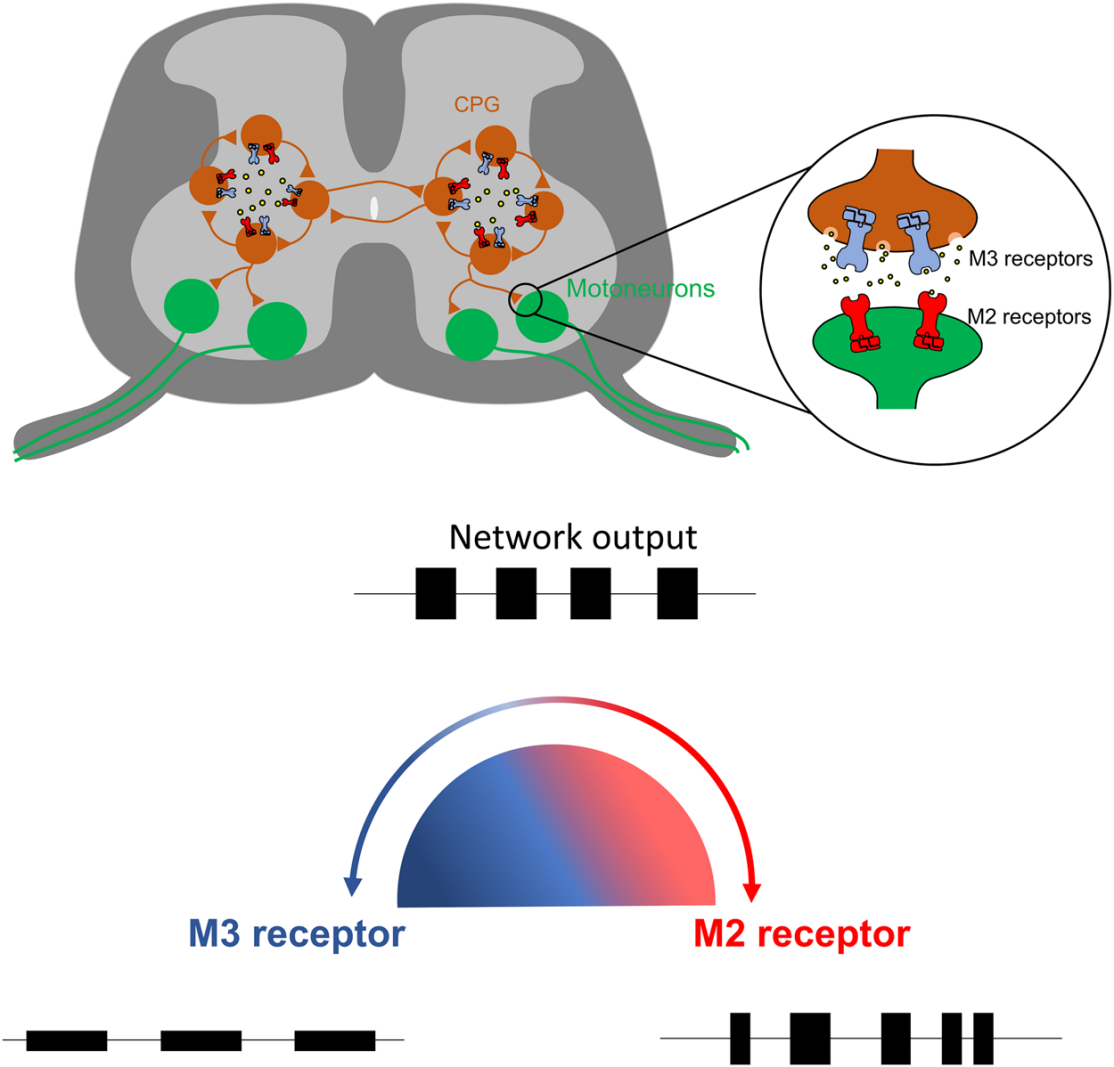
Schematic showing how shifts in the balance between M2 and M3 receptor activation can affect motor output. During rhythmic locomotion acetylcholine (yellow) is released and modulates CPG neurons (orange) as well as motoneurons (green) through M2 (red) and M3 receptor (blue) activation. A fine balance between M2 and M3 receptor activation ensures adequate locomotor patterns, however a decrease in M3 receptor activation (red) disrupts the locomotor pattern whereas a reduction of M2 receptor activation (blue) slows the rhythm and decreases burst amplitude.

Interestingly, our findings at the network and single cell levels reveal that acetylcholine can exhibit opposing modulatory actions on spinal motor networks due to the activation of different muscarinic receptor subtypes, presumably linked to contrasting intracellular signaling pathways with different protein targets. Recently, opposing responses to muscarine in zebrafish motoneurons have been associated with the activation of M2 and non-M2 receptors, a feature suggested to contribute to functional flexibility in the zebrafish swimming circuitry^69^. Such contrasting actions upon spinal locomotor networks, which depend on the receptor subtypes activated, have also been shown for other neuromodulators, such as 5-HT^70,71^, DA^72^ and noradrenalin^73^. Our data support that acetylcholine released from spinal sources during locomotor network activity may typically lead to balanced M2 and M3 receptor activation, which in turn ensures appropriate locomotor output. This ‘balance’ is revealed by our finding that blockade of individual subtypes of muscarinic receptors (M2 vs M3) alters the locomotor rhythm and pattern (Fig. 8). Similar results, including disrupted patterns of locomotion, have been observed when potentiating endogenous levels of acetylcholine via administration of acetylcholinesterase inhibitors^26,74^. Changes between disorganized patterns of activity, and rhythmic, patterned activity are known to relate to appropriate network excitability, which can be balanced by neuromodulators through concentration-dependent activation of different receptor subtypes^75,76^. It is therefore likely that the herein reported opposing effects of M2 and M3 receptors on the spinal CPG help prevent the network from going into an “over-modulated” state to both ensure appropriately patterned locomotor output^41^ and help prevent excitotoxic damage. Given the potential involvement of acetylcholine signaling in spinal cord injury^26^ and ALS^77–79^, our data further highlight the clinical importance of understanding the contributions of cholinergic systems to the control of motor networks.

Our report of dual effects of M2 and M3 receptors in the spinal cord shed new light on the role of intraspinal acetylcholine in mammalian motor systems. We suggest that modulation by cholinergic interneurons through muscarinic receptors is required for appropriate assembly of motor patterns, in addition to task-dependent modulation of the strength of motor output^14^. Besides forming premotor synapses to modulate motoneuron output, acetylcholine-releasing interneurons also strongly modulate the CPG kernel, exerting a local modulation that can facilitate episodes of locomotor-like activity^26^. In the visual and auditory cortices, cholinergic systems are involved in attentional modulation, which enhances responses to stimuli^80,81^. Cholinergic Pitx2^+^ interneurons can be seen as an intraspinal motor counterpart to these supraspinal cholinergic systems, in the sense that C-boutons contribute to task-appropriate changes in the responsiveness of motoneurons to a variety of inputs^14^. Further analyses, focused on the organization and function of specific populations of spinal cholinergic interneurons, will be important to provide additional insight into the roles of intraspinal cholinergic modulatory systems.

## Supplementary information

Supplementary information

## Data Availability

The datasets generated and analysed during the current study are available from the corresponding author upon reasonable request.
