# Supplementary material for: Balanced cholinergic modulation of spinal locomotor circuits via M2 and M3 muscarinic receptors
Source: Sci Rep. 2019 Oct 1;9:14051. doi: 10.1038/s41598-019-50452-1 (PMC6773880; doi:10.1038/s41598-019-50452-1)
Supplement: Supplementary file 1 — Supplementary information [file 41598_2019_50452_MOESM1_ESM.pdf]

Supplementary information

Balanced cholinergic modulation of spinal locomotor circuits via M2 and M3 muscarinic receptors

Filipe Nascimento, Lennart R. B. Spindler & Gareth B. Miles*

School of Psychology and Neuroscience, University of St Andrews,

St Andrews KY16 9JP, United Kingdom

* Correspondence:

Gareth B. Miles

gbm4@st-andrews.ac.uk

+44 (0)1334 463281

School of Psychology & Neuroscience

St Mary’s Quad

St Andrews, Fife

KY16 9JP

United Kingdom

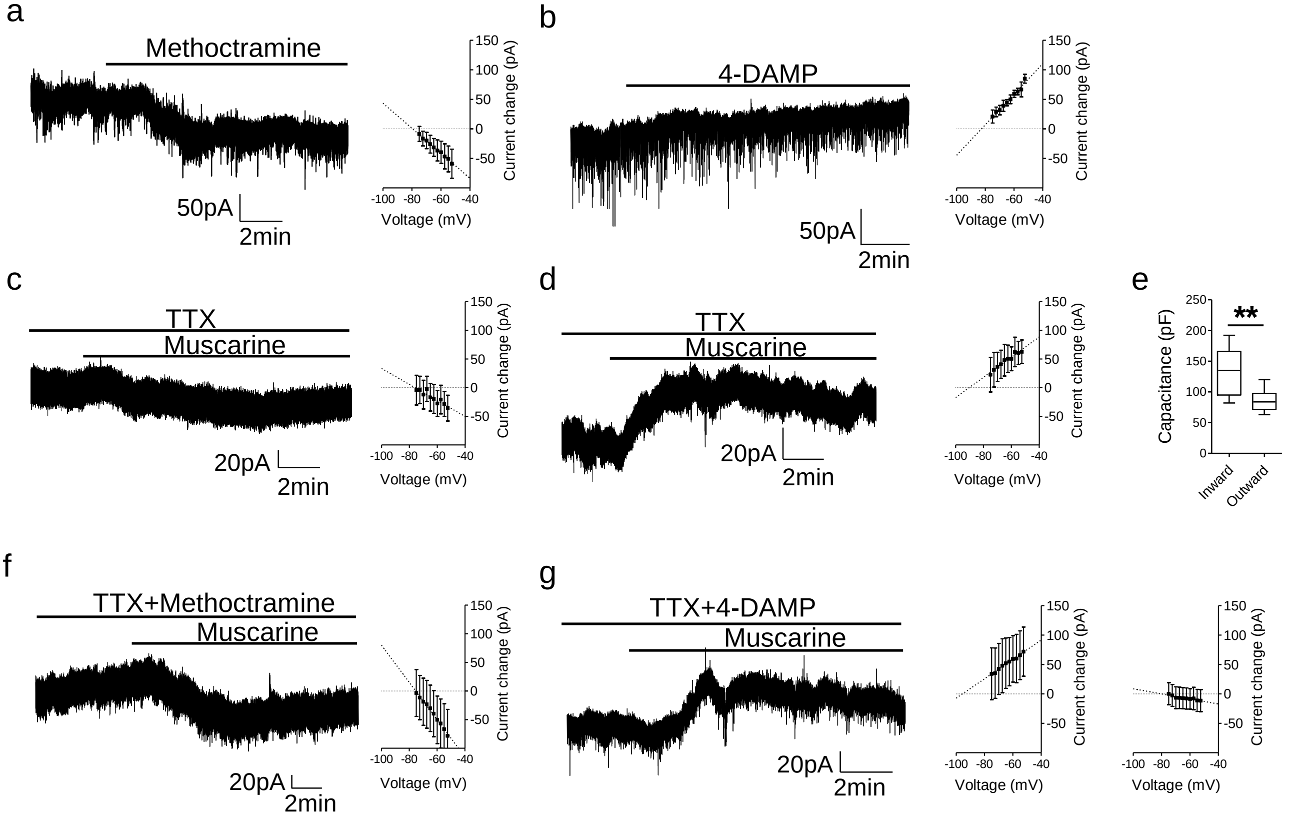

Supplementary figure 1 – M2 receptor antagonist induces an inward current and the M3 receptor blocker an outward current in motoneurons. Voltage clamp recordings and respective I-V plots depicting **(a)** an inward current and increase in input resistance (n=8) elicited by methoctramine and **(b)** outward current and increase in input resistance (n=7) induced by 4-DAMP. **(c)** Representative trace illustrating the inward current and **(d)** outward current elicited by muscarine in the presence of TTX with respective I-V plots depicting an increase (n=6) and decrease (6) in input resistance. **(e)** Plot of average capacitance values for motoneurons exhibiting inward (n=11) versus outward (n=12) currents in response to muscarine co-applied with TTX. **(f)** Illustrative trace of the inward current elicited by muscarine in the presence of methoctramine and TTX with respective I-V plot (n=8). **(g)** Representative trace of the outward current induced by muscarine co-applied with 4-DAMP and TTX and I-V plots for motoneurons that showed an increase (left, n=6) or decrease in input resistance (right, n=6). All recordings were performed at a holding voltage of -60mV. **p<0.01

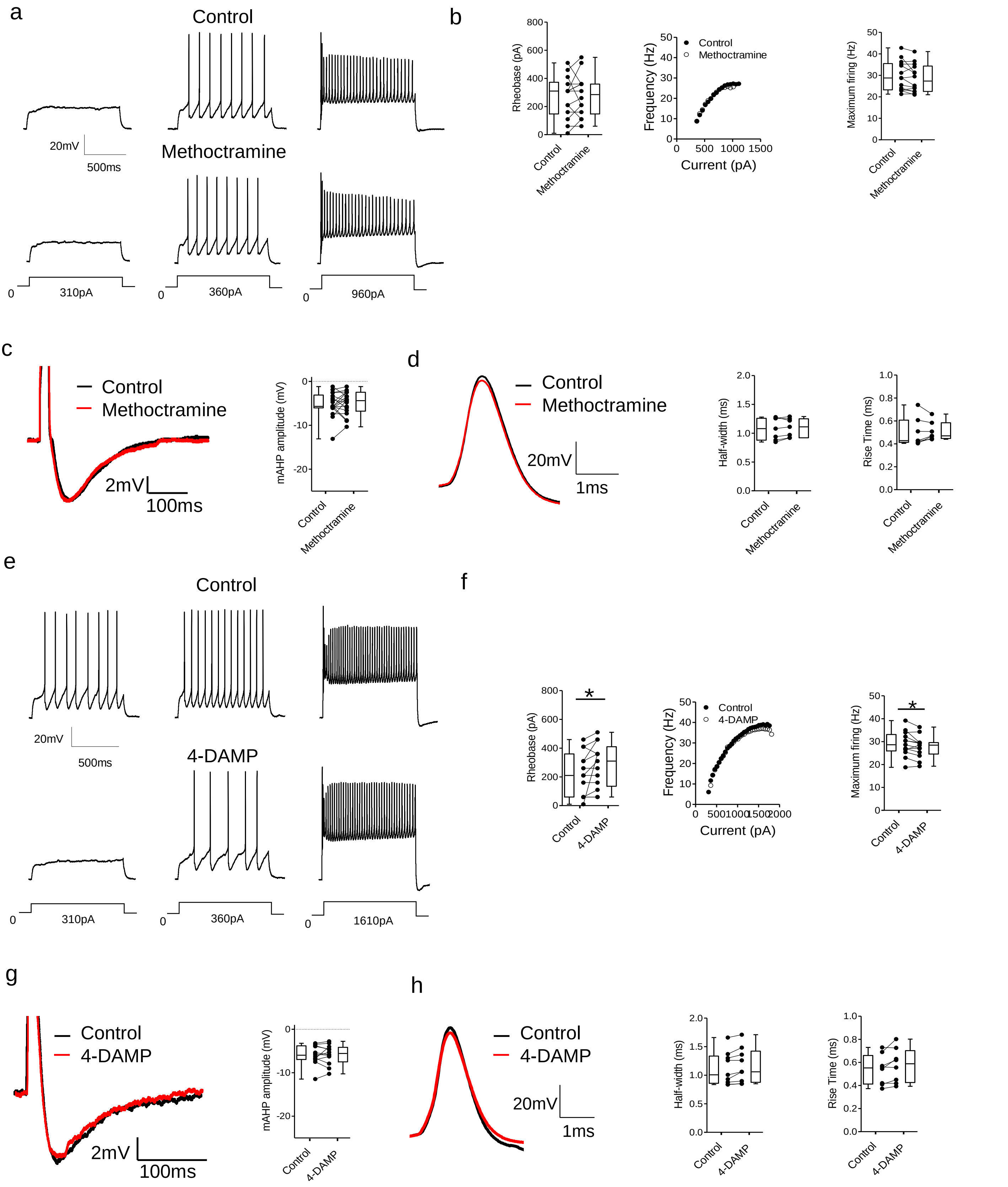
a
Control
Methoctramine
20mV
500ms
0
310pA
360pA
960pA
b
Rheobase (pA)
Control
Methoctramine
Frequency (Hz)
Current (pA)
Maximum firing (Hz)
c
Control
Methoctramine
2mV
100ms
mAHP amplitude (mV)
d
20mV
1ms
Half-width (ms)
Rise Time (ms)
e
Control
4-DAMP
1610pA
f
*
g
h

Supplementary figure 2 – M2 receptor antagonist has no effect on motoneuron firing and M3 receptor blockade decreases firing rate. **(a)** Current-clamp recordings showing the effects of methoctramine on motoneuron firing in response to current injection. **(b)** Graphs showing the effects of methoctramine on rheobase (n=14), an illustrative f-I relationship and maximum firing frequencies (n=18). **(c)** Truncated single action potentials showing the effects of methoctramine on the mAHP (n=17). **(d)** Superimposed action potentials recorded from a motoneuron before and after methoctramine with plots of average values of action potential half-width and rise-time in each condition (n=7). **(e)** Motoneuron firing in response to current injection illustrating the effects of 4-DAMP in the presence of methoctramine. **(f)** Graphs showing the effects of 4-DAMP on rheobase (n=13), an illustrative f-I relationship and maximum firing (n=13). **(g)** Truncated single action potentials illustrating the effect of 4-DAMP on the mAHP (n=12). **(f)** Representative traces of action potentials in the presence of 4-DAMP with plots of average half-width and rise-time (n=9). $^*p<0.05$
